# Correction to: ETHYLENE INSENSITIVE3/EIN3-LIKE1 modulate *FLOWERING LOCUS C* expression via histone demethylase interaction

**DOI:** 10.1093/plphys/kiae358

**Published:** 2024-07-17

This is a correction to: Mengting Xu, Xiaoxiao Li, Wei Xie, Chuyu Lin, Qiannan Wang, Zeng Tao, ETHYLENE INSENSITIVE3/EIN3-LIKE1 modulate *FLOWERING LOCUS C* expression via histone demethylase interaction, Plant Physiology, Volume 192, Issue 3, July 2023, Pages 2290–2300, https://doi.org/10.1093/plphys/kiad131

In the originally published version of this manuscript, there was an error in the accession number for gene IDs. AT2G36270 (FLD), AT3G26790 (EIN3), AT1G21970 (EIL1), AT1G28300 (EIN2) were incorrectly listed. The correct gene IDs are: AT3G10390 (FLD), AT3G20770 (EIN3), AT2G27050 (EIL1), AT5G03280 (EIN2).

The authors sincerely thank the anonymous reader for alerting them to these errors.

The article has been corrected.

